# Antibacterial Properties of Honey Nanocomposite Fibrous Meshes

**DOI:** 10.3390/polym14235155

**Published:** 2022-11-27

**Authors:** Rupy Kaur Matharu, Jubair Ahmed, Jegak Seo, Kersti Karu, Mitra Ashrafi Golshan, Mohan Edirisinghe, Lena Ciric

**Affiliations:** 1 Department of Mechanical Engineering, University College London, Torrington Place, London WC1E 7JE, UK; 2 Department of Civil, Environmental & Geomatic Engineering, University College London, Chadwick Building, Gower Street, London WC1E 6BT, UK; 3 Department of Chemistry, University College London, 20 Gordon Street, London WC1H 0AJ, UK

**Keywords:** antibacterial, fibres, honey, manuka, nanocomposite, composite polymer

## Abstract

Natural substances are increasingly being developed for use in health-related applications. Honey has attracted significant interest, not only for its physical and chemical properties, but also for its antibacterial activity. For the first time, suspensions of Black Forest honeydew honey and manuka honey UMF 20+ were examined for their antibacterial properties against *Escherichia coli* and *Staphylococcus epidermidis* using flow cytometry. The inhibitory effect of honey on bacterial growth was evident at concentrations of 10, 20 and 30 *v*/*v*%. The minimum inhibitory effects of both honey types against each bacterium were also investigated and reported. Electrospray ionisation (ESI) mass spectrometry was performed on both Black Forest honeydew honey and manuka honey UMF 20+. Manuka honey had a gluconic concentration of 2519 mg/kg, whilst Black Forest honeydew honey had a concentration of 2195 mg/kg. Manuka honey demonstrated the strongest potency when compared to Black Forest honeydew honey; therefore, it was incorporated into nanofiber scaffolds using pressurised gyration and 10, 20 and 30 *v*/*v*% manuka honey-polycaprolactone solutions. Composite fibres were analysed for their morphology and topography using scanning electron microscopy. The average fibre diameter of the manuka honey-polycaprolactone scaffolds was found to range from 437 to 815 nm. The antibacterial activity of the 30 *v*/*v*% scaffolds was studied using *S. epidermidis*. Strong antibacterial activity was observed with a bacterial reduction rate of over 90%. The results show that honey composite fibres formed using pressurised gyration can be considered a natural therapeutic agent for various medicinal purposes, including wound-healing applications.

## 1. Introduction

Chronic wounds, such as diabetic foot ulcers, venous leg ulcers and pressure ulcers, are a tremendous burden to wound-care professionals and consume a large quantity of healthcare resources. In the United Kingdom alone, it is estimated that 200,000 patients have chronic wounds and cost the National Healthcare System approximately £3.1 billion per year [1]. Moreover, wounds often become infected, consequently delaying healing, decreasing the patient’s quality of life and affecting recovery. The formation of biofilms can harbour a host of different bacterial species, including both aerobic and anaerobic microorganisms, some of which include *Escherichia coli, Pseudomonas aeruginosa* and *Staphylococcus epidermidis* [2]. However, due to the emergence of antibiotic resistance, topical wound dressings which contain antimicrobial agents have become an important tool for the treatment of infected chronic wounds [3,4,5,6,7]. A multiplicity of antimicrobial agents, particularly, metallic and metallic oxide nanoparticles, have been incorporated into wound dressings and have been clinically evaluated [8,9,10,11,12,13,14,15,16]. Frequently used metallic nanoparticles include silver, gold, copper and zinc [17,18,19,20,21,22,23,24,25,26,27]. However, a major limitation of the use of metallic nanoparticles is detrimental side effects, including argyria and cytotoxicity [28]. This has led to a paradigm shift towards the use of naturally occurring antimicrobial agents [29].

The use of natural materials in a multitude of wound healing applications, from cosmeceuticals to wound dressings, has attracted considerable attention in modern society [1]. Plants play a significant role in traditional and holistic wound healing [2]. The natural active ingredients contained in medicinal plants have immunoregulatory activity, control inflammatory responses, and can affect coagulation, inflammation, epithelialization, collagenization and wound contraction [3,4,5]. A sensible way to maximise the medicinal benefits of these natural materials is to incorporate them into wound dressings. For example, many researchers have incorporated aloe vera into wound dressings to improve the therapeutic effect [6,7,8,9,10]. Additional natural ingredients include cinnamon [11,12,13], lavender [14], cocoa [15], oak bark extracts [16] and others.

Medicinal applications of honey date to ancient Egyptian civilisations. The earliest recording of its use in traditional medicine can be found in the Smith Papyrus, where the prescription of a wound salve is described [30,31,32]. More recently, honey has been used in modern medical practice as a topical agent in the clinical treatment of chronic wounds [33,34,35,36,37,38,39,40,41]. Honey has been shown to accelerate wound healing with effects being noted as early as two days after treatment. In addition, honey has also been shown to encourage epithelisation in sloughs, gangrenous tissue and necrotic tissue, thus minimising the need for surgical intervention [42].

Honey is composed of approximately 200 different substances. Carbohydrates make up the largest constituent of honey (approximately 82–99%), with glucose and fructose representing the main portion [43]. This high sugar concentration plays a major role in the antibacterial mode of action of honey, as well as its acidic pH and hydrogen peroxide production [44,45,46].

In detail, the high osmolarity created by honey’s high sugar and low moisture content draws the cytoplasm out of the bacterial cell and consequently causes shrinking of the cell wall. It has also been suggested that the high osmolarity inhibits microbial growth as the sugar molecules are associated with the water molecules, thus leaving insufficient water for the bacteria to survive [36,47,48]. The acidic pH of honey (due to the gluconic acid in its composition) has been shown to neutralise the alkaline environment of chronic wounds, consequently reducing protease activity, increasing fibroblast activity, and increasing oxygen release [49,50,51], all of which are beneficial during the wound-healing process [51].

However, honeys’ predominant antibacterial mode of action has been attributed to the production of hydrogen peroxide and non-peroxide compounds by glucose oxidase added by bees during its production [44,45,46]. Hydrogen peroxide and non-peroxide compounds act as an antiseptic and stimulate the wound-healing process. Studies have shown that the presence of a hydrogen peroxide gradient causes macrophages to arrive at the wound and release vascular endothelial growth factors and angiogenic factors crucial for wound healing [52,53,54,55]. Honey can continuously provide hydrogen peroxide at a consistent level that is antibacterial and physiologically non-toxic [34]. All three mechanisms are responsible for honey’s healing properties when applied to chronic wounds. Honey’s high sugar content absorbs moisture and establishes a protective layer, thereby preventing microbial penetration and keeping the wound dry [33,56,57,58,59], whilst the acidity assists in the antibacterial action of macrophages, and hydrogen peroxide production keeps the wound sterile [50].

A vast number of papers have been published on the incorporation of honey into fibres for wound dressings; however, the techniques used in these studies yield a low production rate of fibres and, thus, prevent the commercial exploitation of honey in wound-healing applications [60,61,62,63,64,65]. Previous work has reported electrospinning to produce 0.17 kg of fibres per an hour, whilst pressurised gyration can produce 6 kg per an hour [66]. In recent years, pressurised gyration has been utilised for the mass production of uniform nanocomposite fibres [66,67,68,69,70,71,72]. This processing method combines the features of solution blowing and centrifugal spinning to produce large quantities of ultrafine fibres, with tailorable properties. The laboratory setup of pressurised gyration consists of a small aluminium cylindrical vessel with multiple spherical perforations. The vessel itself is attached to an electric motor, capable of speeds of up to 36,000 rpm. High-pressure nitrogen gas (up to 0.3 MPa) is fed into the vessel through the vessel lid. A polymer solution of choice is loaded into the vessel before the apparatus is turned on. In the present study: (i) the antibacterial activity of Black Forest honeydew honey and manuka honey UMF 20+ was determined against two known commonly occurring wound pathogens: *E. coli* and *S. epidermidis;* (ii) the most potent honey was then incorporated into polymeric fibrous meshes using pressurised gyration; (iii) the antibacterial properties of the composite meshes were assessed, along with differences in their morphology. Here we present an industrially scalable approach to manufacturing honey fibre meshes for wound healing. For the first time, the antibacterial activity of Black Forest honeydew honey, the production of honey nanocomposite fibres using pressurised gyration, and the antibacterial activity of the formed pressurised gyrated fibres is reported.

## 2. Materials and Methods

### 2.1. Materials

#### 2.1.1. Honey

Black Forest honeydew honey was purchased from Bulgarian Bee (Okorsh, Bulgaria). Manukora manuka honey UMF 20+ was obtained from Manukora (Auckland, New Zealand). Both honeys were stored in a dry cupboard that was regularly temperature managed (21–24 °C) to prevent crystallisation.

#### 2.1.2. Chemical Analysis

Fifty percent gluconic acid solution (>50% in water) was purchased from Sigma-Aldrich (Gillingham, UK). LC-MS grade water, acetonitrile (CH_3_CN, >99.8%) and formic acid (CH_2_O_2_, >98.0%) were purchased from Sigma-Aldrich (Gillingham, UK).

#### 2.1.3. Bacteria Strains and Media

Antibacterial activity was assessed against *Staphylococcus epidermidis* NCTC 11047 and *Escherichia coli* K12. LB Broth Base (Lennox L Broth Base) was purchased from Sigma-Aldrich (Gillingham, UK). A LIVE/DEAD BacLight Bacterial Viability and Counting Kit were purchased from ThermoFisher Scientific (Paisley, UK). Media were prepared as instructed by the manufacturer.

#### 2.1.4. Polymer Solution Preparation

Chloroform (CAS: 67-66-3) and polycaprolactone (PCL) (mn 80,000) were purchased from Sigma-Aldrich (Gillingham, UK). All solvents and chemicals were of analytical grade and used as received. A polymer solution of 15% (*w*/*v*) PCL in chloroform was prepared. Manuka honey was added to the PCL solution to make 30 *v*/*v*% honey/PCL solutions. The solution mixture was then subjected to high-speed mixing (SpeedMixer DAC 150.1 FVZ-K, Germany) at 3600 rpm for 5 min; care was taken to prevent the solution from heating as this would cause solidification of the honey.

### 2.2. Methods

#### 2.2.1. Chemical Analysis

A range of gluconic acid standard solutions was prepared by diluting a stock solution with water to make 0, 1, 5, 10, 15, and 20 mg/L solutions. Black Forest honeydew honey and manuka honey UMF 20+ were purchased online and stored at room temperature. The samples were diluted with water 100-fold (*w*/*v*). The pH of the honey samples was adjusted to approximately 10.5 by addition of 1 M NaOH to hydrolyse gluocono-δ-lactone to gluconic acid; then, the pH was adjusted to approximately 7.8 with HCl [73]. The pH-adjusted samples were filtered through a 0.45-μm syringe membrane filter (PES; STARLAB LTD, Milton Keynes, United Kingdom).

LC-MS analyses were performed using an Accela LC chromatograph connected to a linear ion trap mass spectrometer (Thermo Fisher Scientific, Oxford, UK). Chromatographic separation was achieved using an Accucore Vanquish C18 column 50 mm × 2.1 mm, 1.5 μm (Thermo Fisher Scientific, Oxford, UK). The C18 column was kept at 30 °C. The mobile phases were: (A) water, 0.1% formic acid and (B) acetonitrile, 0.1% formic acid. The flow rate was set at 200 μL/min. The gradient was as follows: after 1 min at 99% A, the proportion of A% was increased to 95% A over the next 9 mins, then in 6 s B% was changed to 99% and maintained at 99% B for a further 4 mins and 54 s, before returning to 99% A in 6 s re-equilibrating the C18 column for a further 3 min 54 s, giving a total run time of 18 min. The injection volume was 20 μL. The effluent from the C18 column was directed to the electrospray source of the linear trap quadrupole (LTQ) mass spectrometer operated in a positive mode. The LTQ mass spectrometer was set up as follows: spray voltage, 4500 V; capillary temperature, 280 °C; sheath gas pressure, 40 psi; ion sweep gas pressure, 0 psi; auxiliary gas pressure, 5 psi; and skimmer offset value, 25 V. The mass spectrometer was set to scan in a full mode from *m*/*z* 50–500 and in MSMS set at the *m*/*z* 197 with isolation width 2.0 at collision energy 20. Samples were measured in triplicate and the average (± confidence interval) was calculated for the results. The blanks (H_2_O) were analysed between every honey sample.

The calibration curve was constructed from 0 to 20 mg/L of gluconic acid. We evaluated linearity (R^2^), limit of detection (LOD), and limit of quantification (LOQ). The LOD and LOQ were determined using a lowest concentration of analyte 50 ng/L.

#### 2.2.2. Mesh Production

**Figure 1:**
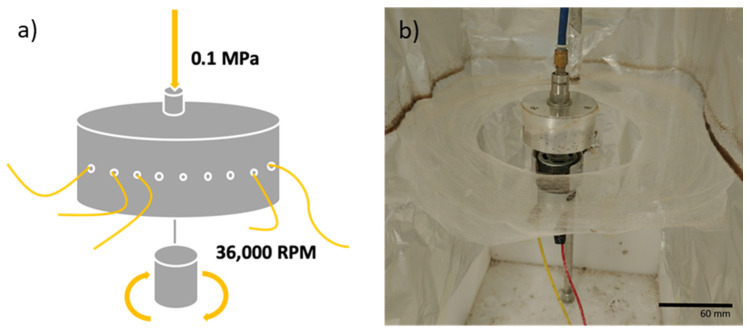
(**a**) diagrammatic representation of the pressurised gyration setup showing gas infusion pressure and maximum rotation speed used; fibres are formed as a product of the two acting forces (**b**) Photograph showing honey-PCL composite fibres surrounding the pressurised gyration vessel, ready to be collected.

The solutions were subjected to spinning via pressurised gyration at a gas flow pressure of 0.1 MPa and a rotational speed of 36,000 rpm. The fibres formed a ring around the gyration vessel, which resembled large bandage-like scaffolds Figure 1. The fibres were then collected via sterile utensils and stored for characterisation and antibacterial studies. The fibre manufacturing process was operated at ambient conditions (22 ± 2 °C, 45–55% relative humidity).

#### 2.2.3. Characterisation

##### Viscosity

The viscosity of the solutions was measured using a Brookfield Viscometer DV-III (Brookfield, Middleboro, MA, USA). A small-sample spindle was used with a polymer volume of 3 mL. For the manuka honey readings, the lowest spindle speed was selected (0.01 rpm); due to the high viscosity range of these solutions, measurements were taken at a constant torque value to ensure comparability within the samples. All viscosity measurements were taken at ambient conditions (22 ± 2 °C) and repeated 3 times to give an average value. The viscosity of the pure manuka honey could not be determined as it fell above the viscosity range of the apparatus; for this reason, we have assumed it is larger than 60,000 mPa s.

##### Surface Tension

The interfacial surface tension of the prepared solutions was characterized via a tensiometer (Tensiometer K9, Kruss GmbH, Hamburg, Germany). The Du Nouy ring method was employed to measure the surface tension. A glass vial was filled with polymer solution and a platinum-iridium ring with a 6 cm diameter was submerged into the solution. The ring in the solution was raised to enable a fluid meniscus to form; the variation of forces was measured using a force tensiometer. These readings were repeated 5 times to find the average surface tension values for each solution of varying concentration.

##### Fibre Morphology

The virgin PCL fibres and the honey composite fibres were characterised for their diameter and their surface topography. The samples, which were gold sputter-coated (Q150R ES, Quorum Technologies) for 90 s prior to imaging at various high magnifications, were examined by scanning electron microscopy (SEM) (Hitachi S-3400n, Chiyoda City, Japan). The SEM images were then surveyed using Image J 1.52q software (National Institutes of Health); 100 fibre strands were measured at random, and the mean diameter was calculated. The frequency distribution of the fibre diameters was calculated using OriginPro 2021 (9.8 SR0) graphical software.

##### Fourier Transform Infrared Spectroscopy

Fourier transform infrared spectroscopy (FTIR) was carried out on a PerkinElmer Spectrum-2 FTIR Spectrophotometer (PerkinElmer Inc., Beaconsfield, UK). The infrared spectra of the fibres and the honey were taken in transmittance mode between the wavenumbers of 4000 to 450 cm^−1^. All measurements were taken at ambient temperature and conditions. For each sample, four scans were taken at a resolution of 4 cm^−1^. The spectra were analysed with essential FTIR software and plotted into graphs using OriginPro 2021 (9.8 SR0).

#### 2.2.4. Antibacterial Activity of Honey and Honey Fibre Meshes

The antibacterial activities of manuka honey and Black Forest honeydew honey were determined against *S. epidermidis* and *E. coli*. Stock cultures of these microorganisms were stored in LB broth supplemented with 10% glycerol at −80 °C until use. The antibacterial activities of the honeys were determined by evaluating the survival of the bacteria strains after incubation with 10, 20 and 30 *v*/*v*% of honey for 24 h at 37 °C and 150 rpm. Honey concentrations of 10, 20 and 30 *v*/*v*% were chosen, as previous studies have shown the minimum inhibitory concentration of honey to range from 4.2 to 25% [74,75].

Overnight *S. epidermidis* and *E. coli* cultures were added to sterile LB broth at 10 *v*/*v*%. Manuka honey or Black Forest honeydew honey was added to the cultures at either 10, 20 or 30 *v*/*v*% and incubated for 24 h at 37 °C and 150 rpm. Post incubation, flow cytometry was used, together with the LIVE/DEAD BacLight Bacterial Viability and Counting Kit to quantify the proportion of live and dead bacteria cells in the suspensions. This method relies on the use of fluorescent stains, SYTO^®^9 and propidium iodide (PI). SYTO^®^9 is a green fluorescent nucleic dye which can penetrate both live and dead cells, whilst PI is a red fluorescent intercalating stain which can only penetrate cells with damaged membranes (non-viable cells) to displace the SYTO^®^9. Therefore, viable cells appear green, whilst non-viable cells have a red colouration. A stock solution of PI and SYTO^®^9 was prepared according to the manufacturers’ instructions. A quantity of 180 µL of the stock staining solution was added to 20 µL of diluted sample and incubated at ambient temperature in the dark for 15 min.

Sample acquisition was performed using a Guava easyCyte^®^ flow cytometer (Merck, Poole, UK) and InCyte software. Regions of interest (gates) were marked out using positive (media and bacteria only), negative (media only) and fluorescent-minus-one controls (single-stained positive controls). A total of 50,000 events were collected. Bacteria acquisition gates were identified using forward-scatter and side-scatter channels. The gated bacteria population was then examined using fluorescent channels to identify live and dead cell populations. FlowJo was used to gate the live and dead bacterial cell counts where the proportions of live and dead bacteria cells were calculated. All experiments were repeated at least three times.

The minimum inhibitory concentration (MIC) of both Black Forest honeydew honey and manuka honey UMF 20+ was also investigated. Both honeys were diluted with molecular grade water to produce the concentration range. The highest concentration of both diluted honeys was 50 *v*/*v*% and the lowest concentration was 5 *v*/*v*% (50, 45, 40, 35, 30, 25, 20, 15, 10, 5 *v*/*v*%, and control). Each diluted honey sample was mixed thoroughly by vortexing at maximum speed for 30 s. The samples were covered with aluminium foil to prevent UV-light damage on the chemicals in the samples and kept in a −20 °C freezer.

The microdilution MIC of Black Forest honeydew honey and manuka honey was determined against *E. coli* and *S. epidermidis*. To prepare the bacterial stock solution, *E. coli* and *S. epidermidis* were added to 15 mL of sterile LB broth for 24 h at 37 °C and 150 rpm. After incubation, absorbance of each stock solution was diluted to 0.015 nm at OD_600_ using photometry. A quantity of 12 mL of the diluted solution was prepared for use in a microdilution MIC test. The method reported by Wiegand et al. (2008) for assessment of the possible growth patterns in MIC microtiter plates was followed [76]. This method is accredited by the EUCAST MIC database. A total of 4 technical replicates and 2 biological replicates were conducted for each bacterium. A FLUOstar Omega Microplate Reader (BMG LabTech, Ortenberg, Germany) was used.

The prepared honey-PCL composite fibrous meshes were tested for antibacterial activity against *S. epidermidis* and *E. coli*. Overnight cultures were added to sterile LB broth at 10 *v*/*v*%. To this, honey-PCL scaffolds were added at 10 wt% and incubated for 24 h at 37 °C and 150 rpm. Pure PCL fibrous scaffolds were used as the negative control. The number of live bacteria in the suspension post exposure was enumerated using flow cytometry.

#### 2.2.5. Statistical Analysis

The antibacterial activity of both honeys was statistically analysed and compared using a one-way ANOVA with a post hoc Tukey’s honest significant differences (HSD) test. The antibacterial activity of the prepared honey-PCL fibres was also statistically analysed and compared to the control fibres using a one-way ANOVA with a post hoc Tukey HSD test. The difference was considered significant when *p* < 0.05.

## 3. Results and Discussion

### 3.1. Chemical Analysis

**Figure 2:**
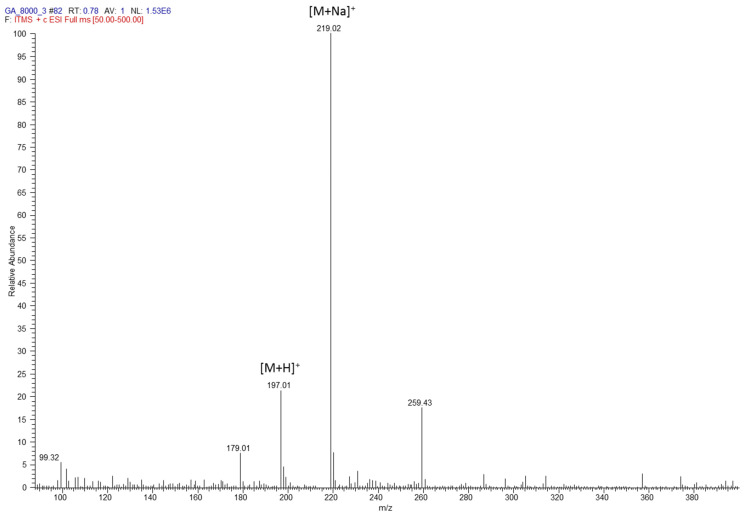
ESI mass spectrum of gluconic acid in 1 mg/L in water, 0.1% formic acid acquired by a direct injection of this solution into the LTQ mass spectrometer at a flow rate 20 µL/min.

Gluconic acid is the most abundant acid in honey. Various studies have demonstrated the important role gluconic acid has in the antibacterial activity of honey [75,77,78]. Gluconic acid has the empirical formula C_6_H_12_O_7_ and an exact mass of 196.0577. Figure 2 shows the ESI mass spectrum of gluconic acid. The peak at *m*/*z* 197.01 corresponds to [M+H]^+^ ions formed during ESI ionization. The peak at *m*/*z* 219 corresponds to [M+Na]^+^ ions. The loss of water from gluconic acid was also observed corresponding to the peak at *m*/*z* 179 [M-H_2_O]^+^. MSMS was performed on *m*/*z* 197 was carried out to confirm the structure of gluconic acid. The identification of gluconic acid in honey was based on its chromatographic retention time, ESI mass spectrum and MSMS spectrum. MSMS of *m*/*z* 197 generated fragment ions at *m*/*z* values of 74, 82, 114 and 122 (Supplementary Information Figures S1–S3).

**Figure 3:**
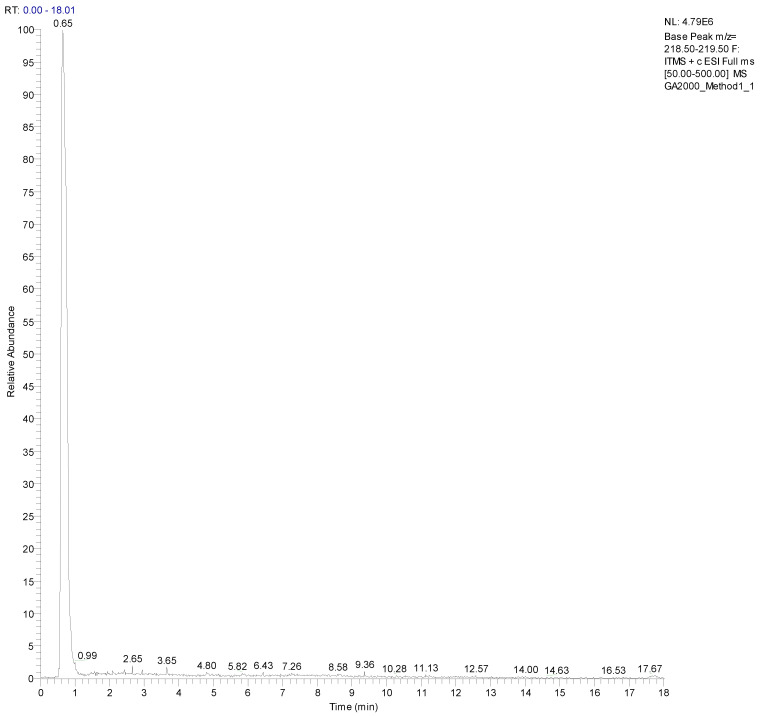
Reconstructed ion chromatogram of *m*/*z* 219, [M+Na]^+^ ions corresponding to gluconic acid from Manuka honey sample.

The identification of gluconic acid in honey samples was based on its chromato-graphic retention time, ESI mass spectrum and MSMS spectrum. The reconstructed ion chromatograms were constructed for *m*/*z* 219 [M+Na]^+^ corresponding to gluconic acid, which shows that the authentic gluconic acid eluted from the C18 column at 0.65 min (Figure 3). Also, a single chromatographic peak was observed at the retention time of 0.65 min in the honey samples and its ESI and MSMS spectra were identical to the authentic gluconic acid.

In Table 1, the calibration curve was constructed for gluconic acid using the linear range concentration (1, 5, 10, 15, and 20 mg/L); the coefficient of determination (R^2^) was 0.999. The LOD and LOQ were calculated using the calibration curve. The LOD was 0.82 mg/kg and the LOQ was 2.49 mg/kg. The concentration of gluconic acid was expected to be higher than the LOD and LOQ.

Each honey sample was tested in triplicate and the peak area was measured to calculate the concentration using an external calibration. The average measured concentrations and standard deviation were calculated for each honey to determine the concentration of gluconic acid in the honey samples (Table 2). By comparing with the existing literature related to the gluconic acid concentration in honey [79], a similar concentration of gluconic acid with the same type of honey was observed.

### 3.2. Antibacterial Activity of Honey

**Figure 4:**
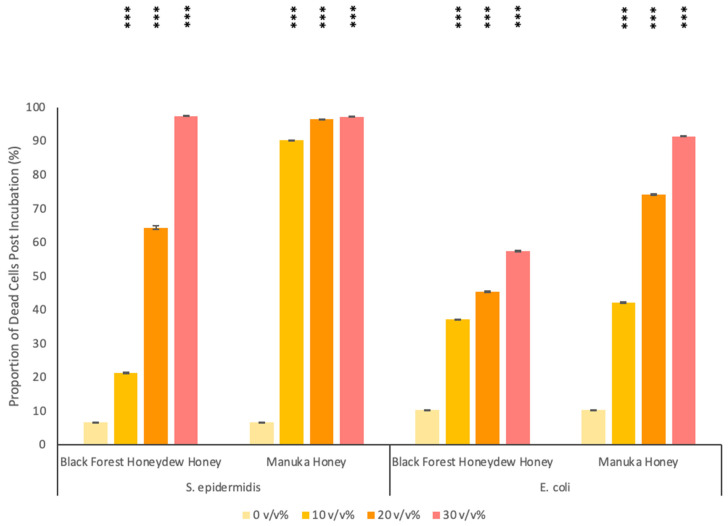
Antibacterial activity of S. epidermis and E. coli against Black Forest honeydew and manuka honey at concentrations of 10, 20 and 30 (*v*/*v*%). The antibacterial efficiency is given here as the percentage proportion of dead cells following incubation. *p* values of < 0.001 (***) when compared to the control are shown on the graph.

The sensitivity of *S. epidermidis* and *E. coli* towards Black Forest honeydew honey and manuka honey was assessed. As shown in Figure 4, the antibacterial activity of both types of honey was concentration-dependent, with the most potent effects being noted at higher honey volume concentrations. For both Gram-positive *S. epidermidis* and Gram-negative *E. coli*, the manuka honey performed better when compared with the Black Forest honeydew honey.

For *S. epidermidis*, Black Forest honeydew honey exhibited potent antibacterial activity at 30 *v*/*v*% (97.4 ± 0.026% of the bacterial population were dead after incubation), whilst manuka honey was very effective at all three concentrations tested (>90% cell death post incubation). For *E. coli*, Black Forest honeydew honey showed mild cytotoxic properties, as only 37.1 ± 0.068%, 45.3 ± 0.243% and 57.4 ± 0.169% of the bacterial population were dead after incubation, with 10, 20 and 30 *v/v*% of honey, respectively. When compared to honeys from apiarists and honey-packers, Black Forest honeydew honey exhibited stronger antibacterial activity against *S. epidermidis*, as these honeys did not exhibit antibacterial activity at 75 *v*/*v*% [80]. However, when compared to Tualang honey, the antibacterial properties of Black Forest honeydew honey were not as prominent. Tan et al. reported that Tualang honey resulted in 95% growth inhibition at a concentration of 22.5% [81]. Manuka honey had antibacterial properties at 30 *v*/*v*% as 91.4 ± 0.069% of the bacteria cell population were found dead after incubation; this finding is similar to what has previously been reported in the literature [81].

The MIC results further corroborated these findings. For *S. epidermidis*, the MIC was 30 *v*/*v*% and 20 *v*/*v*% with Black Forest honeydew honey and manuka honey, respectively. There was no difference between the MIC results and the flow cytometry results which showed a nearly 100% proportion of dead cells. For *E. coli*, the MIC was 45 *v*/*v*% and 40 *v*/*v*% for Black Forest honeydew honey and manuka honey, respectively. The MIC results were higher than the highest concentration tested using flow cytometry. At a concentration of 30 *v/v*%, more than 10% of the cell population survived; therefore, it was expected the MIC values would be higher than 30 *v*/*v*%.

The differences in antibacterial activity between the two types of honey tested are thought to be the result of differences in their chemical composition. As reported in this study, manuka honey has a slightly higher concentration of gluconic acid, when compared to Black Forest honeydew honey. Additionally, although the antibacterial activity of honey is generally multifaceted, the antibacterial activity of Black Forest honeydew honey is primarily caused by hydrogen peroxide, polyphenolic compounds and the interaction between the two [46]. In honey, hydrogen peroxide is produced by the glucose-oxidase-mediated conversion of glucose to gluconic acid under aerobic conditions [82]. Therefore, the high concentration of glucose oxidase in Black Forest honeydew honey plays an important role in the generation of hydrogen peroxide and its antibacterial activity [46,83]. In addition, the polyphenolic compounds in Black Forest honeydew honey act as pro-oxidants when in the presence of transition metal ions and peroxides. The polyphenolic compounds work in two ways to accelerate antibacterial activity: (i) by directly producing hydrogen peroxide; and (ii) by reducing iron, which triggers the Fenton reaction to create more potent reactive oxygen species, such as hydroxyl radicals. It has also been shown that the chemical interaction of honey polyphenols with hydrogen peroxide results in the generation of products responsible for the degradation of bacterial DNA [84,85]. The synergistic effect of polyphenols and hydrogen oxide has been shown to be more effective against bacteria than hydrogen peroxide alone and can induce oxidative stress-related responses in bacteria [86].

Whilst hydrogen peroxide and polyphenolic compounds were the major factors involved in the bactericidal activity of Black Forest honeydew honey, these factors are present in relatively low concentrations in manuka honey. Methylglyoxal is thought to play a major role in the antibacterial activity of manuka honey. Manuka honey contains a hundred-fold higher concertation of methylglyoxal when compared to conventional honeys [87,88,89]. Methylglyoxal is formed non-enzymatically by the conversion of nectar-derived dihydroxyacetone, which is present at exceptionally high concentrations in the nectar of manuka trees [88,89]. Methylglyoxal is a reactive metabolite that can exert toxic effects by inhibiting protein and DNA synthesis by reacting with guanine residues in RNA/DNA and its precursors [90,91,92]. A plethora of previous literature reports have demonstrated methylglyoxal to be the sole antibacterial agent in manuka honey [87,89,93].

Overall, both honeys were more potent towards Gram-positive *S. epidermidis* than Gram-negative *E. coli*. The difference in potency is attributed to the difference in cell wall structure. Though Gram-positive bacteria have a thicker multi-layered cell wall, they have no outer membrane and lipopolysaccharide content, making them more vulnerable to antimicrobial agents. The one-way ANOVA showed an overall significant difference for all treatments. The p-value (1.1102 × 10^–16^) corresponding to the F-statistic of one-way ANOVA was lower than 0.01, suggesting that one or more treatments were significantly different. The post hoc Tukey HSD results showed that all treatments, when compared to the control, were statistically significant (Tukey HSD *p*-value = 0.001 and Tukey HSD inference = *p* < 0.01).

### 3.3. Honey Composite Fibrous Meshes

Solution rheology and other solution characteristics can often explain and determine the formation of fibres and their consequential structure. Table 3 displays the viscosity measurements of the created polymer solutions together with the surface tension. These numbers are important in differentiating the different solution concentration uptakes, but also in indicating how their rheology will affect the manipulation of the fluid within the gyration vessel.

Honey is known to be one of the most viscous naturally occurring edible products [94]. In this study, honey composite fibres were made by combining 15% PCL polymer solutions with manuka honey and, thus, were high in viscosity. Virgin PCL had the lowest viscosity at 7638 mPa.S. With the addition of 10% *v/v* manuka honey, the viscosity increased over five-fold from the virgin PCL value to 44,091 mPa s; the increase in viscosity is ascribed to the requirement of a higher shear force at a given shear rate, as in gyratory deformation. The manuka honey used in these experiments fell above the range of the equipment used; therefore, it is understood that the viscosity was over 60,000 mPa s. In the cases shown above, all the composite solutions displayed extremely high levels of viscosity, with such solution behaviour not having been previously explored in a gyration-based manufacturing process.

The surface tension of a polymer solution plays a pivotal role in polymer jet formation for electrically and centrifugally based fibre-forming techniques; this polymer jet subsequently dries to give rise to fibrous structures [95,96]. Table 1 shows the recorded surface tension values for the adopted solutions. It was found that the surface tension of both the PCL and manuka honey solutions were very closely matched. This explains the small deviation in number for the composite solutions; all solutions possessed a very similar value for surface tension. From these measured values, we can conclude that the centrifugal force from the rotation-pressurised gyration vessel could readily overcome the interfacial surface tension of all the tested solutions and that the viscosity of the solutions plays a key role in fibre production and the resulting morphology and structure that is typically seen [1,2].

**Figure 5:**
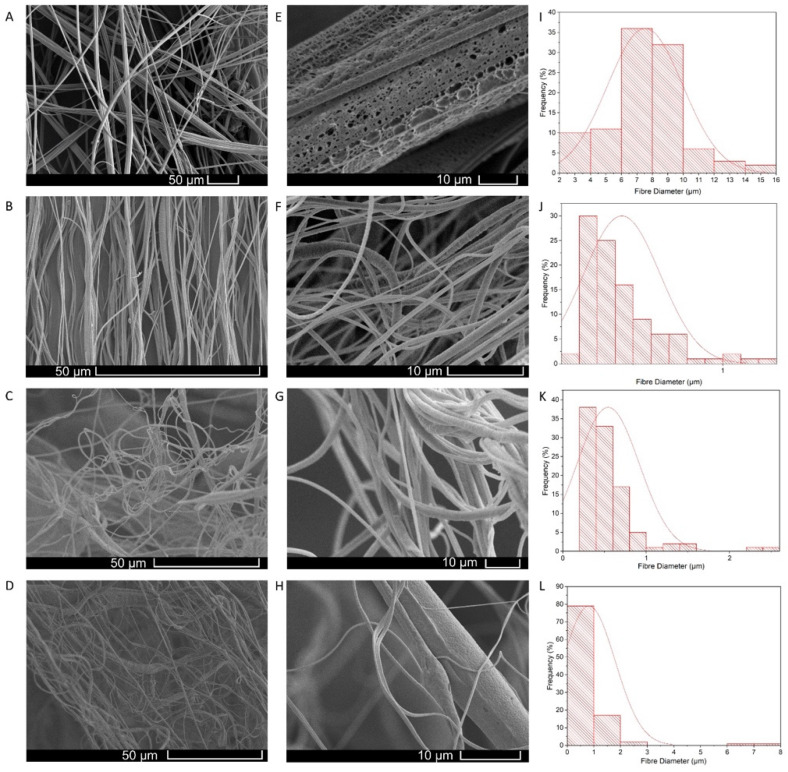
Scanning Electron Microscope Image of Fibres Produced. (**A**) Virgin PCL fibres, (**B**) 10% honey-composite fibres, (**C**) 20% honey-composite fibres, (**D**) 30% honey-composite fibres. High magnification images of (**E**) virgin PCL fibres, (**F**) 10% honey-composite fibres, (**G**) 20% honey-composite fibres, (**H**) 30% honey-composite fibres, along with corresponding fibre diameter distribution histograms (**I**–**L**).

The solutions were spun with the gyration setup and their morphology was analysed using SEM. The micrographs of all the produced and tested samples are presented in Figure 5.

The respective polymer solutions were used to spin the fibres; the fibre-forming process utilised here, like electrospinning and other techniques, relies heavily on solvent evaporation [97]. The resulting fibres differed in morphology due to many features of the original solution, such as solvent volatility, viscosity, polymer chain entanglement and surface tension [98,99,100,101]. The micrographs of the mentioned samples are presented in Figure 5. The average fibre diameter for the virgin PCL fibres was 7.5 ± 2.4 μm; these fibre diameters coincide with typical values from PCL-based fibres at molecular weights of over 60,000 mn [102]. Due to the high viscosity of the PCL solution and the high molecular weight (80,000 mn), the polymer solution had a high degree of polymer chain entanglement which led to relatively thick fibres being produced. As reported by Croisier et al. the Young’s moduli of the PCL fibrous scaffolds was 3.8 ± 0.8 MPa [103]. The average diameter for the 10% honey composite fibres was 437 ± 21 nm. This steep and sudden drop in fibre diameter does not coincide with normal trends observed, where an increase in polymer viscosity leads to thicker formed fibres [104]. It is not fully understood what caused the large reduction in fibre diameter; however, very high viscosity solutions differ in rheology to lower solutions. It is assumed that the thick solution reduced the orifice aperture of the gyration pot and subsequently caused thinner fibres to extrude from the reduced orifice.

The average diameter for the 20% honey composite fibres was 543 ± 374 nm. It is evident that the change from 10–20% *w*/*w* honey caused a noticeable increase in the fibre diameter. This was expected as the honey concentration is increased and, thus, the fibres contain more of the thick and viscous medium. The average diameter for the 30% honey composite fibres was 815 ± 98 nm; again, the increase in honey content and the viscosity of the solution resulted in another sizable increase in fibre thickness. These fibres were, however, still very thin and, thus, can afford a very high surface-area-to-volume ratio which is highly suitable for a wide range of biomedical applications [105]. In a wound environment, thinner fibres can more closely resemble the surrounding extracellular matrix and provide a suitable niche for fibroblasts and other pivotal cells involved in the wound response [106,107,108,109]. At 30 *v/v*%, there is a trade-off between the fibre diameter and the honey concentration. For the initial honey antibacterial tests, it was found that 30% manuka honey killed over 90% of both Gram-positive and Gram-negative bacteria. For these reasons, 30% manuka-PCL scaffolds were chosen as the optimal antibacterial structures to be tested.

High magnification images of the fibre surface revealed the surface topography of the scaffolds in more detail. The fibres had nanopores which were visible on their surface. The presence of nanopores arises from the use of a volatile solvent (chloroform) which causes condensation through temperature change as it evaporates; these condensation droplets then dry and, in their place, leave surface pores [110]. The presence of surface pores confirms the evaporation of chloroform. These pores further increase the available surface area of the fibres and can even contain pockets of manuka honey that can trap small microbes and release active ingredients (such as antimicrobial peptides from the honey) as a function of time.

**Figure 6:**
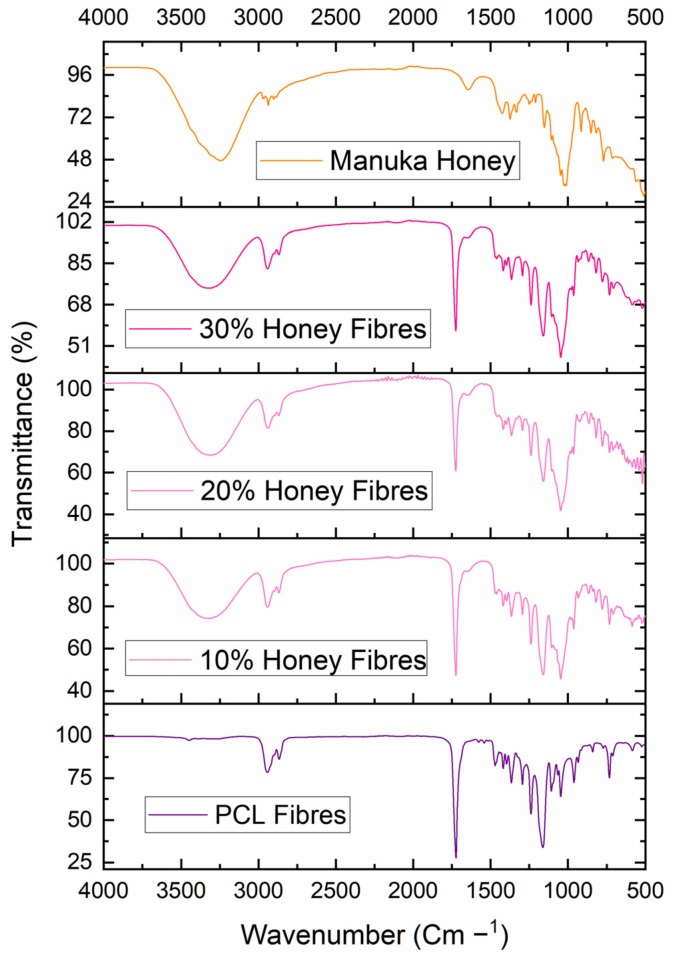
FTIR spectra of virgin PCL fibres, 30% honey-loaded fibres and of pure manuka honey. Specific peaks associated with manuka honey and with PCL are highlighted using arrows in the composite fibres.

The structures of the virgin PCL, the manuka honey and the honey-loaded fibres were analysed using FTIR to confirm the presence and uptake of honey into the fibrous scaffold (Figure 6). The virgin PCL fibres showed characteristic peaks at 2950 cm^−1^, 1725 cm^−1^ and 1165 cm^−1^. The peak at 2950 cm^−1^ is linked to asymmetric CH_2_ stretching, the peak at 1165 cm^−1^ is related to symmetric COC stretching, and the peak at 1725 cm^−1^ corresponds to the carbonyl vibration region of PCL [111,112]. Pure manuka honey shows characteristic bands at wavelengths of 3240 cm^−1^, 1645 cm^−1^ and 1020 cm^−1^_._ The band that peaked at around 3240 cm^−1^ is likely to belong to the O-H stretching group of the honey; this peak can cover a broader range than the others because of the intramolecular bond type [113,114]. At a wavelength of 1645 cm^−1^, the peak probably corresponds to a carbonyl bond group from the honey, while the peak at 1020 cm^−1^ is likely to correlate to the C-O and C-O stretching vibrations of honey [115]. The absorbance profiles of all the honey-loaded fibres were similar, so only 30% manuka fibre profiles are shown in the spectrum. It can be seen that, by overlaying the spectrum of the manuka fibres, there is an overlap of the prominent peaks at around 3220–3360 cm^−1^ between the manuka honey and the honey fibres. This peak is due to the O-H bonds in the manuka honey and appears only in the presence of honey in this spectrum, confirming the uptake of manuka honey in the PCL fibres. Additionally, the two PCL-containing samples showed a characteristic band at 2950 cm^−1^, confirming the presence of CH_2_ stretching that is typically found in the polymer. The honey fibres and the pure manuka honey profiles overlap at wavenumbers of around 1055 cm^−1^, confirming the presence of honey in the fibres.

As manuka honey was found to have the strongest antibacterial properties in this study, it was incorporated into the polymeric fibres at a concentration of 30% *v/v*. Composite fibres with 30% honey had the largest diameter, but were also the most uniform of the fibres tested. There was a tight distribution of the fibre diameter, which can be beneficial in producing bandage-like structures to ensure that the final product meets strict conditions in the manufacturing process.

During the fibre production process, 4 mL of honey-PCL solution took 10 s to process into fibres. During this time, an average of 0.4 g was produced in each run. As this process relies on solvent manipulation, yield loss is mostly attributed to solvent evaporation. Theoretically, and based on the current results, if the production technique was scaled up and run continuously, it would be capable of outputting over 8.6 kg/hour of nanofibres with the same small 60 mm gyration vessel; this value is much larger than that for a single setup of any electrospinning device [116]. As the gyration setup also takes very little floor space, the yield can be improved by increasing the number of gyration pots and the volume of the polymer solution. In contrast to electrospinning, multiple gyration setups can be in operation simultaneously, as electrical interference between neighbouring pots is not a concern [117].

### 3.4. Antibacterial Studies of Honey Composite Fibres

**Figure 7:**
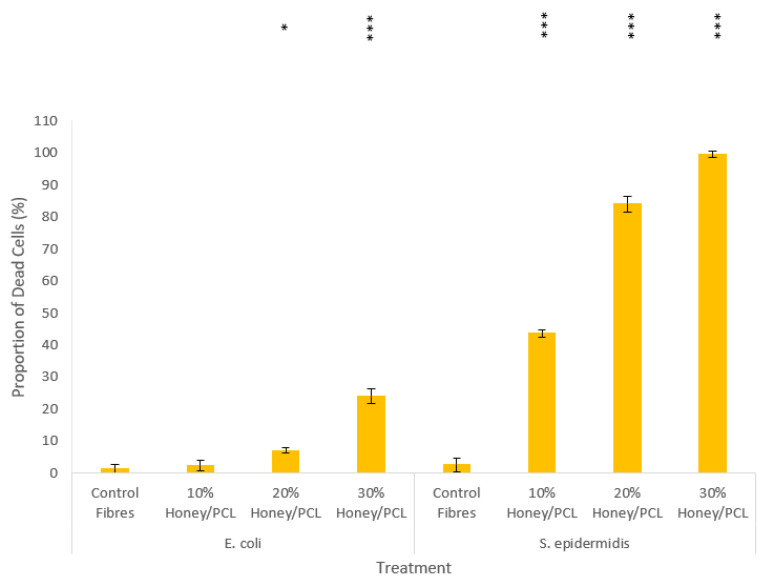
Antibacterial activity of 10, 20 and 30% manuka-PCL composite fibrous meshes compared to the negative control of virgin PCL fibres; antibacterial effectiveness is expressed as the percentage bacterial reduction. The post hoc Tukey HSD results of the treatments compared to the control are shown on the graph as *p* values of < 0.05 (*) and <0.001 (***).

The antibacterial activity of the 30 *v/v*% manuka honey composite fibres were tested against *S. epidermidis* and is shown in Figure 7. The honey-PCL meshes were effective against Gram-positive *S. epidermidis* compared with the negative control (which did not show any antibacterial activity, *p* = 0.0041). The scaffolds were able to reduce the bacterial concentration by 93.2 ± 0.1%. This high bacterial reduction highlights the strong antibacterial activity of the composite fibres at only 30 *v/v*%. PCL has not been previously documented as possessing antibacterial capability and showed no sign of bacterial reduction, as was expected. The antibacterial activity of the honey-PCL meshes was similar to what has been observed with silver-impregnated wound dressings, where microbial reductions of >90% after 24 h have been reported [118]. The fibres tested were spun at 36,000 rpm with a flow pressure of 0.1 MPa and were able to carry the honey whilst retaining a high level of antibacterial activity, which was not lost in the solution-making process. Though the manuka honey composite fibres were effective against *E. coli*, the antibacterial efficacy was lower compared to pure honey. While the same proportion of manuka honey was used, some of the manuka honey may have been entrapped inside the fibre and, therefore, not exposed on the fibre surface. This would have affected the antimicrobial activity as the microbial cells are inevitably exposed to a lower concentration of honey. The one-way ANOVA on the manuka-PCL composite fibrous meshes against *E. coli*, showed that all concentrations, excluding the 10 *v/v*% honey-PCL meshes, were significantly different (F statistic = 117.39 and *p*-value = 5.9358 × 10^–7^). The one-way ANOVA on the manuka-PCL composite fibrous meshes against *S. epidermidis* was considered significantly significant as the *p*-value (1.2929 × 10^–11^) corresponding to the F-statistic (1758.4367) was lower than 0.05.

*S. epidermidis* infections are becoming increasingly prevalent in hospital environments, partly due to the high density of immunocompromised patients [119]. Although not typically pathogenic, *S. epidermidis* represents a concern due to its tendency to form biofilms [120]. Moreover *S. epidermidis* can function as a gene reservoir, which allows genes to be transferred to *Staphylococcus aureus* which can lead to the enhancement of its antibiotic resistance [121]. As *S. epidermidis* is part of the skin microbiome, the honey-composite fibrous meshes provide a protective layer by means of the fibrous scaffold; the honey comes in contact with the bacterial niche and can significantly reduce the population. The antimicrobial effect against the Gram-negative *E. coli* was less pronounced, likely due to the difference in the cell wall structure, as discussed above.

**Figure 8:**
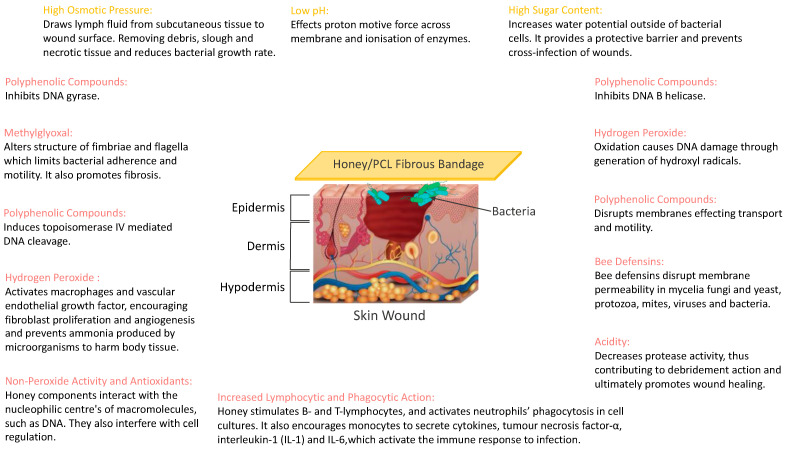
Antimicrobial mechanisms of Honey/PCL bandages during wound healing.

As described before, the mechanism of manuka honeys’ anti-bacterial action is thought to involve a combination of acidic pH regulation and osmotic effects on bacterial cells [34]. As a wound-healing additive, honey is also thought to possess anti-inflammatory ability, which allows for cleaner healing and has been shown to stimulate immune responses [122]. Cells integral to the wound-healing process, such as tumour necrosis factor-alpha, interleukin-1beta and interleukin-6, have been shown in in vitro studies using manuka honey to be released at increased levels, which may account for the improved healing observed [106,123]. Figure 8 shows a schematic illustration of the antimicrobial mechanism of the prepared honey/PCL fibres.

In fibre form, honey is significantly easier to store, package and apply evenly to a wound. The PCL fibres in these composites allow for the even distribution of honey within the fibre surface and the polymer matrix. As a biodegradable material, PCL can degrade as a function of time, releasing honey over long periods of time, resulting in the need for fewer bandage applications and reducing the risk of trauma from the removal process. In addition, the honey in the fibres can draw wound exudate to the fibres through osmosis, thus keeping the wound dry. Furthermore, the fibres produced here demonstrate sub-micrometre thickness, providing a large surface area for cell attachment during the wound-healing process. It has been reported that the application of honey over a wound often causes a stinging pain to the patient; however, by incorporating honey into a fibrous scaffold, the honey content is vastly reduced, whilst retaining a high kill rate, and, therefore, may help to mitigate the pain. Cytotoxicity of the prepared fibres is of no concern as both honey and PCL are routinely used in the medical field as topical treatments [124,125].

## 4. Conclusions

In this study, Black Forest honeydew honey and manuka honey UMF 20+ were demonstrated to have enormous potential as antibacterial agents. Both honeys exhibited strong antibacterial properties against *S. epidermidis*, with a cell death rate of >90% post incubation being observed. When considering *E. coli*, manuka honey UMF 20+ performed better than Black Forest honeydew honey, with a maximum microbial reduction of 91.4% being achieved. This was probably due to a higher concertation of gluconic acid in its composition. Novel composite manuka honey-PCL fibrous meshes were successfully prepared by pressurised gyration. SEM images showed the fibres to be uniform, with fibre diameters ranging from 437 to 815 nm. The antibacterial properties of the prepared composite fibres were tested against common skin-related hospital-acquired bacterial strains. It was observed that the 30% composite fibres reduced the bacterial concentration by over 93%. The composite scaffolds were produced by a highly scalable manufacturing technology with high production ability, which should be capable of producing fibres at a rate of over 8 kg/h with a single setup. We have demonstrated a successful route to the mass production of honey fibrous meshes which can be incorporated in bandages. These have the benefit of high antibacterial activity with the advantages of low honey usage and high surface area.

## Figures and Tables

**Table 1:** Linear ranges, linear equation, the coefficient of determination (R2), and limit of detection (LOD) and quantification (LOQ) for gluconic acid.

Organic Acid	Linear Range (mg/kg)	Linear Equation	R^2^	LOD (mg/L)	LOQ (mg/L)
**Gluconic acid**	1–20	*f(x)* = 1387315x + 15875058	0.999	0.05	0.49

**Table 2:** Peak area, measured concentration (mg/kg), average concentration (mg/kg), and ±confidence interval (C.I.) for the triplicates of honey samples.

Honey (Replicates)	Peak Area	Measured Concentration (mg/kg)	Average Concentration (mg/kg)	±C.I.
**Manuka honey (1)**	50,125,757	2468.8	2519.2	80.9
**Manuka honey (2)**	52,118,205	2612.5		
**Manuka honey (3)**	50,227,315	2476.2		
**Black forest honey (1)**	46,298,596	2193.0	2195.2	7.1
**Black forest honey (2)**	44,439,647	2059.0		
**Black forest honey (3)**	46,251,523	2189.6		

**Table 3:** Viscosity and surface tension of the studied polymer solutions and manuka honey. In all cases, the PCL solution consisted of a 15% (*w*/*v*) concentration. The solution represents the ratio of honey to PCL solution.

Solution	Viscosity (mPa s)	Surface Tension (mN m^−1^)
100% Virgin PCL	7638 ± 128	33.1 ± 1.1
15% Virgin PCL	7638 ± 128	33.1 ± 1.1
10% Honey/PCL	44,091 ± 56	33.6 ± 1.1
20% Honey/PCL	49,265 ± 89	34.4 ± 1.2
30% Honey/PCL	54,096 ± 69	35.1 ± 0.8
100% Manukora Manuka Honey	>60,000	32.5 ± 1.6

## Supplementary Materials

The following supporting information can be downloaded at: https://www.mdpi.com/article/10.3390/polym14235155/s1, Figure S1: Black Forest honey samples (triplicates): reconstructed ion chromatogram for *m/z* 219 [M+Na]^+^; Figure S2: Manuka honey samples (triplicates): reconstructed ion chromatogram for *m/z* 219 [M+Na]^+^; Figure S3: A calibration curve of diluted gluconic acid standard (0, 1, 5, 10, 15, 20 mg/L).

## References

[1] Posnett J., Franks P.J. (2008). The Burden of Chronic Wounds in the UK. Nurs. Times.

[2] Yang L., Liu Y., Wu H., Høiby N., Molin S., Song Z.j. (2011). Current understanding of multi-species biofilms. Int. J. Oral Sci..

[3] Lipsky B.A., Hoey C. (2009). Topical antimicrobial therapy for treating chronic wounds. Clin. Infect. Dis..

[4] Coutts P., Sibbald R.G. (2005). The effect of a silver-containing Hydrofiber dressing on superficial wound bed and bacterial balance of chronic wounds. Int. Wound J..

[5] Ousey K., McIntosh C. (2009). Topical antimicrobial agents for the treatment of chronic wounds. Br. J. Community Nurs..

[6] Boni B.O.O., Lamboni L., Bakadia B.M., Hussein S.A., Yang G. (2020). Combining Silk Sericin and Surface Micropatterns in Bacterial Cellulose Dressings to Control Fibrosis and Enhance Wound Healing. Eng. Sci..

[7] Brako F., Luo C., Matharu R.K., Ciric L., Harker A., Edirisinghe M., Craig D.Q.M. (2020). A Portable Device for the Generation of Drug-Loaded Three-Compartmental Fibers Containing Metronidazole and Iodine for Topical Application. Pharmaceutics.

[8] Jones S.A., Bowler P.G., Walker M., Parsons D. (2004). Controlling wound bioburden with a novel silver-containing Hydrofiber dressing. Wound Repair Regen..

[9] Cutting K., White R., Edmonds M. (2007). The safety and efficacy of dressings with silver—Addressing clinical concerns. Int. Wound J..

[10] Borkow G., Gabbay J., Dardik R., Eidelman A.I., Lavie Y., Grunfeld Y., Ikher S., Huszar M., Zatcoff R.C., Marikovsky M. (2010). Molecular mechanisms of enhanced wound healing by copper oxide-impregnated dressings. Wound Repair Regen..

[11] Lansdown A.B., Mirastschijski U., Stubbs N., Scanlon E., Agren M.S. (2007). Zinc in wound healing: Theoretical, experimental, and clinical aspects. Wound Repair Regen..

[12] Matharu R.K., Ciric L., Edirisinghe M. (2018). Nanocomposites: Suitable alternatives as antimicrobial agents. Nanotechnology.

[13] Martinotti S., Ranzato E. (2018). Honey, Wound Repair and Regenerative Medicine. J. Funct. Biomater..

[14] Li S., Jasim A., Zhao W., Fu L., Ullah M.W., Shi Z., Yang G. (2018). Fabrication of pH-electroactive Bacterial Cellulose/Polyaniline Hydrogel for the Development of a Controlled Drug Release System. ES Mater. Manuf..

[15] Xie S., Zhang X., Walcott M.P., Lin H. (2018). Applications of Cellulose Nanocrystals: A Review. Eng. Sci..

[16] Nair S.S., Zhu J.Y., Deng Y., Ragauskas A.J. (2014). Hydrogels Prepared from Cross-Linked Nanofibrillated Cellulose. ACS Sustain. Chem. Eng..

[17] Li S., Dong S., Xu W., Tu S., Yan L., Zhao C., Ding J., Chen X. (2018). Antibacterial Hydrogels. Adv. Sci..

[18] Wu J., Zheng Y., Wen X., Lin Q., Chen X., Wu Z. (2014). Silver nanoparticle/bacterial cellulose gel membranes for antibacterial wound dressing: Investigation in vitro and in vivo. Biomed. Mater..

[19] Rujitanaroj P.-O., Pimpha N., Supaphol P. (2008). Wound-dressing materials with antibacterial activity from electrospun gelatin fiber mats containing silver nanoparticles. Polymer.

[20] Shalumon K., Anulekha K., Nair S.V., Nair S., Chennazhi K., Jayakumar R. (2011). Sodium alginate/poly (vinyl alcohol)/nano ZnO composite nanofibers for antibacterial wound dressings. Int. J. Biol. Macromol..

[21] Kumar P., Lakshmanan V.-K., Biswas R., Nair S.V., Jayakumar R. (2012). Synthesis and biological evaluation of chitin hydrogel/nano ZnO composite bandage as antibacterial wound dressing. J. Biomed. Nanotechnol..

[22] Li Q., Lu F., Zhou G., Yu K., Lu B., Xiao Y., Dai F., Wu D., Lan G. (2017). Silver inlaid with gold nanoparticle/chitosan wound dressing enhances antibacterial activity and porosity, and promotes wound healing. Biomacromolecules.

[23] Lu B., Ye H., Shang S., Xiong Q., Yu K., Li Q., Xiao Y., Dai F., Lan G. (2018). Novel wound dressing with chitosan gold nanoparticles capped with a small molecule for effective treatment of multiantibiotic-resistant bacterial infections. Nanotechnology.

[24] Baghaie S., Khorasani M.T., Zarrabi A., Moshtaghian J. (2017). Wound healing properties of PVA/starch/chitosan hydrogel membranes with nano Zinc oxide as antibacterial wound dressing material. J. Biomater. Sci. Polym. Ed..

[25] Ahmed R., Tariq M., Ali I., Asghar R., Khanam P.N., Augustine R., Hasan A. (2018). Novel electrospun chitosan/polyvinyl alcohol/zinc oxide nanofibrous mats with antibacterial and antioxidant properties for diabetic wound healing. Int. J. Biol. Macromol..

[26] Simões D., Miguel S.P., Ribeiro M.P., Coutinho P., Mendonça A.G., Correia I.J. (2018). Recent advances on antimicrobial wound dressing: A review. Eur. J. Pharm. Biopharm..

[27] Rajendran N.K., Kumar S.S.D., Houreld N.N., Abrahamse H. (2018). A review on nanoparticle based treatment for wound healing. J. Drug Deliv. Sci. Technol..

[28] Lansdown A.B.G. (2010). Silver in Healthcare: Its Antimicrobial Efficacy and Safety in Use.

[29] Ahmed J., Altun E., Aydogdu M.O., Gunduz O., Kerai L., Ren G., Edirisinghe M. (2019). Anti-fungal bandages containing cinnamon extract. Int. Wound J..

[30] Zumla A., Lulat A. (1989). Honey—A remedy rediscovered. J. R. Soc. Med..

[31] Al-Jabri A.A. (2005). Honey, milk and antibiotics. Afr. J. Biotechnol..

[32] Bansal V., Medhi B., Pandhi P. (2005). Honey—A remedy rediscovered and its therapeutic utility. Kathmandu Univ. Med. J..

[33] Efem S.E. (1988). Clinical observations on the wound healing properties of honey. Br. J. Surg..

[34] Lusby P.E., Coombes A., Wilkinson J.M. (2002). Honey: A potent agent for wound healing?. J. Wound Ostomy Cont. Nurs..

[35] Bergman A., Yanai J., Weiss J., Bell D., David M.P. (1983). Acceleration of wound healing by topical application of honey. An animal model. Am. J. Surg..

[36] Al-Waili N., Salom K., Al-Ghamdi A.A. (2011). Honey for wound healing, ulcers, and burns; data supporting its use in clinical practice. ScientificWorldJournal.

[37] Oryan A., Zaker S.R. (1998). Effects of topical application of honey on cutaneous wound healing in rabbits. J. Vet. Med. Ser. A.

[38] Subrahmanyam M. (1991). Topical application of honey in treatment of burns. Br. J. Surg..

[39] Carreck N.L. (2018). Special issue: Honey. J. Apic. Res..

[40] Samarghandian S., Farkhondeh T., Samini F. (2017). Honey and Health: A Review of Recent Clinical Research. Pharmacogn. Res..

[41] Meo S.A., Al-Asiri S.A., Mahesar A.L., Ansari M.J. (2017). Role of honey in modern medicine. Saudi J. Biol. Sci..

[42] Cavanagh D., Beazley J., Ostapowicz F. (1970). Radical operation for carcinoma of the vulva. A new approach to wound healing. J. Obstet. Gynaecol. Br. Commonw..

[43] Ball D.W. (2007). The Chemical Composition of Honey. J. Chem. Educ..

[44] Bizerra F.C., Da Silva P.I., Hayashi M.A. (2012). Exploring the antibacterial properties of honey and its potential. Front. Microbiol..

[45] Alsomal N.A., Coley K.E., Molan P.C., Hancock B.M. (1994). Susceptibility of Helicobacter-Pylori to the Antibacterial Activity of Manuka Honey. J. R. Soc. Med..

[46] Bucekova M., Buriova M., Pekarik L., Majtan V., Majtan J. (2018). Phytochemicals-mediated production of hydrogen peroxide is crucial for high antibacterial activity of honeydew honey. Sci. Rep..

[47] Kumar K.P.S., Bhowmik D., Chiranjib Biswajit Chandira M.R. (2010). Medicinal uses and health benefits of Honey: An Overview. J. Chem. Pharm. Res..

[48] Chirife J., Scarmato G., Herszage L. (1982). Scientific basis for use of granulated sugar in treatment of infected wounds. Lancet.

[49] Khan F.R., Abadin Z.U., Rauf N. (2007). Honey: Nutritional and medicinal value. Int. J. Clin. Pract..

[50] Gethin G.T., Cowman S., Conroy R.M. (2008). The impact of Manuka honey dressings on the surface pH of chronic wounds. Int. Wound J..

[51] Calvin M. (1998). Cutaneous wound repair. Wounds.

[52] Yoo S.K., Huttenlocher A. (2009). Innate Immunity: Wounds Burst H_2_O_2_ Signals to Leukocytes. Curr. Biol..

[53] Cho M., Hunt T.K., Hussain M.Z. (2001). Hydrogen peroxide stimulates macrophage vascular endothelial growth factor release. Am. J. Physiol.-Heart Circ. Physiol..

[54] Burdon R.H. (1995). Superoxide and Hydrogen-Peroxide in Relation to Mammalian-Cell Proliferation. Free Radical. Bio. Med..

[55] Jacobs T., Artusa S., Offerman C., Barnes L., McIntosh A. (2015). The Use of 100% Manuka Honey for Moist Wound Management and Promotion of Autolytic Debridement in an Inpatient Population with Wounds of Mixed Etiology. J. Wound Ostomy Cont..

[56] Santos-Buelga C., González-Paramás A.M., Alvarez-Suarez J.M. (2017). Chemical Composition of Honey. Bee Products—Chemical and Biological Properties.

[57] Khan S.U., Anjum S.I., Rahman K., Ansari M.J., Khan W.U., Kamal S., Khattak B., Muhammad A., Khan H.U. (2018). Honey: Single food stuff comprises many drugs. Saudi J. Biol. Sci..

[58] Nolan V.C., Harrison J., Cox J.A. (2019). Dissecting the Antimicrobial Composition of Honey. Antibiotics.

[59] Oryan A., Alemzadeh E., Moshiri A. (2016). Biological properties and therapeutic activities of honey in wound healing: A narrative review and meta-analysis. J. Tissue Viability.

[60] Minden-Birkenmaier B.A., Neuhalfen R.M., Janowiak B.E., Sell S.A. (2015). Preliminary Investigation and Characterization of Electrospun Polycaprolactone and Manuka Honey Scaffolds for Dermal Repair. J. Eng. Fiber Fabr..

[61] Maleki H., Gharehaghaji A.A., Dijkstra P.J. (2013). A novel honey-based nanofibrous scaffold for wound dressing application. J. Appl. Polym. Sci..

[62] Hixon K.R., Lu T., McBride-Gagyi S.H., Janowiak B.E., Sell S.A. (2017). A Comparison of Tissue Engineering Scaffolds Incorporated with Manuka Honey of Varying UMF. Biomed. Res. Int..

[63] Sarhan W.A., Azzazy H.M.E. (2015). High concentration honey chitosan electrospun nanofibers: Biocompatibility and antibacterial effects. Carbohydr. Polym..

[64] Sarhan W.A., Azzazy H.M.E., El-Sherbiny I.M. (2016). Honey/Chitosan Nanofiber Wound Dressing Enriched with Allium sativum and Cleome droserifolia: Enhanced Antimicrobial and Wound Healing Activity. ACS Appl. Mater. Interfaces.

[65] Arslan A., Simsek M., Aldemir S.D., Kazaroglu N.M., Gumusderelioglu M. (2014). Honey-based PET or PET/chitosan fibrous wound dressings: Effect of honey on electrospinning process. J. Biomat. Sci.-Polym. E.

[66] Mahalingam S., Edirisinghe M. (2013). Forming of Polymer Nanofibers by a Pressurised Gyration Process. Macromol. Rapid Comm..

[67] Matharu R.K., Porwal H., Ciric L., Edirisinghe M. (2018). The effect of graphene-poly(methyl methacrylate) fibres on microbial growth. Interface Focus.

[68] Matharu R.K., Charani Z., Ciric L., Illangakoon U.E., Edirisinghe M. (2018). Antimicrobial activity of tellurium-loaded polymeric fiber meshes. J. Appl. Polym. Sci..

[69] Altun E., Aydogdu M.O., Koc F., Crabbe-Mann M., Brako F., Kaur-Matharu R., Ozen G., Kuruca S.E., Edirisinghe U., Gunduz O. (2018). Novel Making of Bacterial Cellulose Blended Polymeric Fiber Bandages. Macromol. Mater. Eng..

[70] Matharu R.K., Ciric L., Ren G., Edirisinghe M. (2020). Comparative Study of the Antimicrobial Effects of Tungsten Nanoparticles and Tungsten Nanocomposite Fibres on Hospital Acquired Bacterial and Viral Pathogens. Nanomaterials.

[71] Matharu R.K., Tabish T.A., Trakoolwilaiwan T., Mansfield J., Moger J., Wu T., Lourenço C., Chen B., Ciric L., Parkin I.P. (2020). Microstructure and antibacterial efficacy of graphene oxide nanocomposite fibres. J. Colloid Interface Sci..

[72] Matharu R.K., Porwal H., Chen B., Ciric L., Edirisinghe M. (2020). Viral filtration using carbon-based materials. Med. Devices Sens..

[73] Mato I., Huidobro J.F., Simal-Lozano J., Sancho M.T. (2006). Rapid determination of nonaromatic organic acids in honey by capillary zone electrophoresis with direct ultraviolet detection. J. Agric. Food Chem..

[74] Boateng J., Diunase K.N. (2015). Comparing the Antibacterial and Functional Properties of Cameroonian and Manuka Honeys for Potential Wound Healing-Have We Come Full Cycle in Dealing with Antibiotic Resistance?. Molecules.

[75] Mandal M.D., Mandal S. (2011). Honey: Its medicinal property and antibacterial activity. Asian Pac. J. Trop. Biomed..

[76] Wiegand I., Hilpert K., Hancock R.E. (2008). Agar and broth dilution methods to determine the minimal inhibitory concentration (MIC) of antimicrobial substances. Nat. Protoc..

[77] Masoura M., Passaretti P., Overton T.W., Lund P.A., Gkatzionis K. (2020). Use of a model to understand the synergies underlying the antibacterial mechanism of H2O2-producing honeys. Sci. Rep..

[78] Bang L.M., Buntting C., Molan P. (2003). The effect of dilution on the rate of hydrogen peroxide production in honey and its implications for wound healing. J. Altern. Complement. Med..

[79] Suto M., Kawashima H., Nakamura Y. (2020). Determination of organic acids in honey by liquid chromatography with tandem mass spectrometry. Food Anal. Methods.

[80] Basualdo C., Sgroy V., Finola M.S., Marioli J.M. (2007). Comparison of the antibacterial activity of honey from different provenance against bacteria usually isolated from skin wounds. Vet. Microbiol..

[81] Tan H.T., Rahman R.A., Gan S.H., Halim A.S., Hassan S.A., Sulaiman S.A., Bs K.-K. (2009). The antibacterial properties of Malaysian tualang honey against wound and enteric microorganisms in comparison to manuka honey. BMC Complement. Altern. Med..

[82] White J.W., Subers M.H., Schepartz A.I. (1963). The identification of inhibine, the antibacterial factor in honey, as hydrogen peroxide and its origin in a honey glucose-oxidase system. Biochim. Biophys. Acta-Spec. Sect. Enzymol. Subj..

[83] Bucekova M., Valachova I., Kohutova L., Prochazka E., Klaudiny J., Majtan J. (2014). Honeybee glucose oxidase—Its expression in honeybee workers and comparative analyses of its content and H2O2-mediated antibacterial activity in natural honeys. Naturwissenschaften.

[84] de Graft-Johnson J., Nowak D. (2017). Effect of Selected Plant Phenolics on Fe^2+^-EDTA-H_2_O_2_ System Mediated Deoxyribose Oxidation: Molecular Structure-Derived Relationships of Anti- and Pro-Oxidant Actions. Molecules.

[85] Brudzynski K., Abubaker K., Miotto D. (2012). Unraveling a mechanism of honey antibacterial action: Polyphenol/H2O2-induced oxidative effect on bacterial cell growth and on DNA degradation. Food Chem..

[86] Liu X.X., Li J.R., Wang Y.B., Li T.T., Zhao J., Zhang C.H. (2013). Green tea polyphenols function as prooxidants to inhibit Pseudomonas aeruginosa and induce the expression of oxidative stress-related genes. Folia Microbiol..

[87] Kwakman P.H.S., Velde A.A.T., de Boer L., Vandenbroucke-Grauls C.M.J.E., Zaat S.A.J. (2011). Two Major Medicinal Honeys Have Different Mechanisms of Bactericidal Activity. PLoS ONE.

[88] Adams C.J., Boult C.H., Deadman B.J., Farr J.M., Grainger M.N.C., Manley-Harris M., Snow M.J. (2008). Isolation by HPLC and characterisation of the bioactive fraction of New Zealand manuka (Leptospermum scoparium) honey. Carbohydr. Res..

[89] Mavric E., Wittmann S., Barth G., Henle T. (2008). Identification and quantification of methylglyoxal as the dominant antibacterial constituent of Manuka (*Leptospermum scoparium*) honeys from New Zealand. Mol. Nutr. Food Res..

[90] Krymkiewicz N., Dieguez E., Rekarte U.D., Zwaig N. (1971). Properties and mode of action of a bactericidal compound (=methylglyoxal) produced by a mutant of Escherichia coli. J. Bacteriol..

[91] Hayashi K., Fukushima A., Hayashi-Nishino M., Nishino K. (2014). Effect of methylglyoxal on multidrug-resistant Pseudomonas aeruginosa. Front. Microbiol..

[92] Kalapos M.P. (2008). The tandem of free radicals and methylglyoxal. Chem.-Biol. Interact..

[93] Atrott J., Henle T. (2009). Methylglyoxal in Manuka Honey—Correlation with Antibacterial Properties. Czech J. Food Sci..

[94] Garcia J.M., Chambers E., Matta Z., Clark M. (2005). Viscosity Measurements of Nectar- and Honey-thick Liquids: Product, Liquid, and Time Comparisons. Dysphagia.

[95] Ahmed J., Matharu R.K., Shams T., Illangakoon U.E., Edirisinghe M. (2018). A Comparison of Electric-Field-Driven and Pressure-Driven Fiber Generation Methods for Drug Delivery. Macromol. Mater. Eng..

[96] Fridrikh S.V., Yu J.H., Brenner M.P., Rutledge G.C. (2003). Controlling the Fiber Diameter during Electrospinning. Phys. Rev. Lett..

[97] De Vrieze S., Van Camp T., Nelvig A., Hagström B., Westbroek P., De Clerck K. (2009). The effect of temperature and humidity on electrospinning. J. Mater. Sci..

[98] Mahalingam S., Pierin G., Colombo P., Edirisinghe M. (2015). Facile one-pot formation of ceramic fibres from preceramic polymers by pressurised gyration. Ceram. Int..

[99] Obregon N., Agubra V., Pokhrel M., Campos H., Flores D., De la Garza D., Mao Y., Macossay J., Alcoutlabi M. (2016). Effect of Polymer Concentration, Rotational Speed, and Solvent Mixture on Fiber Formation Using Forcespinning^®^. Fibers.

[100] Husain O., Lau W., Edirisinghe M., Parhizkar M. (2016). Investigating the particle to fibre transition threshold during electrohydrodynamic atomization of a polymer solution. Mater. Sci. Eng. C.

[101] Zhang X., Lu Y. (2014). Centrifugal spinning: An alternative approach to fabricate nanofibers at high speed and low cost. Polym. Rev..

[102] Altun E., Aydogdu M.O., Crabbe-Mann M., Ahmed J., Brako F., Karademir B., Aksu B., Sennaroglu M., Eroglu M.S., Ren G. (2018). Co-Culture of Keratinocyte-Staphylococcus aureus on Cu-Ag-Zn/CuO and Cu-Ag-W Nanoparticle Loaded Bacterial Cellulose: PMMA Bandages. Macromol. Mater. Eng..

[103] Croisier F., Duwez A.S., Jérôme C., Léonard A.F., van der Werf K.O., Dijkstra P.J., Bennink M.L. (2012). Mechanical testing of electrospun PCL fibers. Acta Biomater..

[104] Nezarati R.M., Eifert M.B., Cosgriff-Hernandez E. (2013). Effects of Humidity and Solution Viscosity on Electrospun Fiber Morphology. Tissue Eng. Part C Methods.

[105] Ramakrishna S., Fujihara K., Teo W.-E., Yong T., Ma Z., Ramaseshan R. (2006). Electrospun nanofibers: Solving global issues. Mater. Today.

[106] Tonks A.J., Cooper R.A., Jones K.P., Blair S., Parton J., Tonks A. (2003). Honey stimulates inflammatory cytokine production from monocytes. Cytokine.

[107] Werner S., Krieg T., Smola H. (2007). Keratinocyte–Fibroblast Interactions in Wound Healing. J. Investig. Dermatol..

[108] Martin P. (1997). Wound Healing—Aiming for Perfect Skin Regeneration. Science.

[109] Schultz G.S., Wysocki A. (2009). Interactions between extracellular matrix and growth factors in wound healing. Wound Repair Regen..

[110] Illangakoon E.U., Mahalingam S., Matharu K.R., Edirisinghe M. (2017). Evolution of Surface Nanopores in Pressurised Gyrospun Polymeric Microfibers. Polymers.

[111] He Y., Inoue Y. (2000). Novel FTIR method for determining the crystallinity of poly (ε-caprolactone). Polym. Int..

[112] Elzein T., Nasser-Eddine M., Delaite C., Bistac S., Dumas P. (2004). FTIR study of polycaprolactone chain organization at interfaces. J. Colloid Interface Sci..

[113] Azam N., Amin K.A.M. (2018). Influence of Manuka Honey on Mechanical Performance and Swelling Behaviour of Alginate Hydrogel Film. Mater. Sci. Eng..

[114] Azam N., Amin K. The physical and mechanical properties of gellan gum films incorporated manuka honey as wound dressing materials. Proceedings of the IOP Conference Series: Materials Science and Engineering.

[115] Kasprzyk I., Depciuch J., Grabek-Lejko D., Parlinska-Wojtan M. (2018). FTIR-ATR spectroscopy of pollen and honey as a tool for unifloral honey authentication. The case study of rape honey. Food Control..

[116] Persano L., Camposeo A., Tekmen C., Pisignano D. (2013). Industrial Upscaling of Electrospinning and Applications of Polymer Nanofibers: A Review. Macromol. Mater. Eng..

[117] Yang E., Shi J., Xue Y. (2010). Influence of electric field interference on double nozzles electrospinning. J. Appl. Polym. Sci..

[118] Bowler P., Jones S., Walker M., Parsons D. (2004). Microbicidal properties of a silver-containing Hydrofiber^®^ dressing against a variety of burn wound pathogens. J. Burn. Care Rehabil..

[119] Vuong C., Otto M. (2002). Staphylococcus epidermidis infections. Microbes Infect..

[120] Heilmann C., Schweitzer O., Gerke C., Vanittanakom N., Mack D., Götz F. (1996). Molecular basis of intercellular adhesion in the biofilm-forming Staphylococcus epidermidis. Mol. Microbiol..

[121] Otto M. (2009). Staphylococcus epidermidis—The ‘accidental’ pathogen. Nat. Rev. Microbiol..

[122] Almasaudi S.B., El-Shitany N.A., Abbas A.T., Abdel-dayem U.A., Ali S.S., Al Jaouni S.K., Harakeh S. (2016). Antioxidant, Anti-inflammatory, and Antiulcer Potential of Manuka Honey against Gastric Ulcer in Rats. Oxidative Med. Cell. Longev..

[123] Visavadia B.G., Honeysett J., Danford M.H. (2008). Manuka honey dressing: An effective treatment for chronic wound infections. Br. J. Oral Maxillofac. Surg..

[124] Jull A.B., Rodgers A., Walker N. (2015). Honey as a topical treatment for wounds. Cochrane Database Syst. Rev..

[125] Ulery B.D., Nair L.S., Laurencin C.T. (2011). Biomedical Applications of Biodegradable Polymers. J. Polym. Sci. B Polym. Phys..

