# Supplementary material for: Antibacterial Properties of Honey Nanocomposite Fibrous Meshes
Source: Polymers (Basel). 2022 Nov 27;14(23):5155. doi: 10.3390/polym14235155 (PMC9740266; doi:10.3390/polym14235155)
Supplement: Supplementary file 1 [file polymers-14-05155-s001.zip › polymers-1702133-supplementary.pdf]

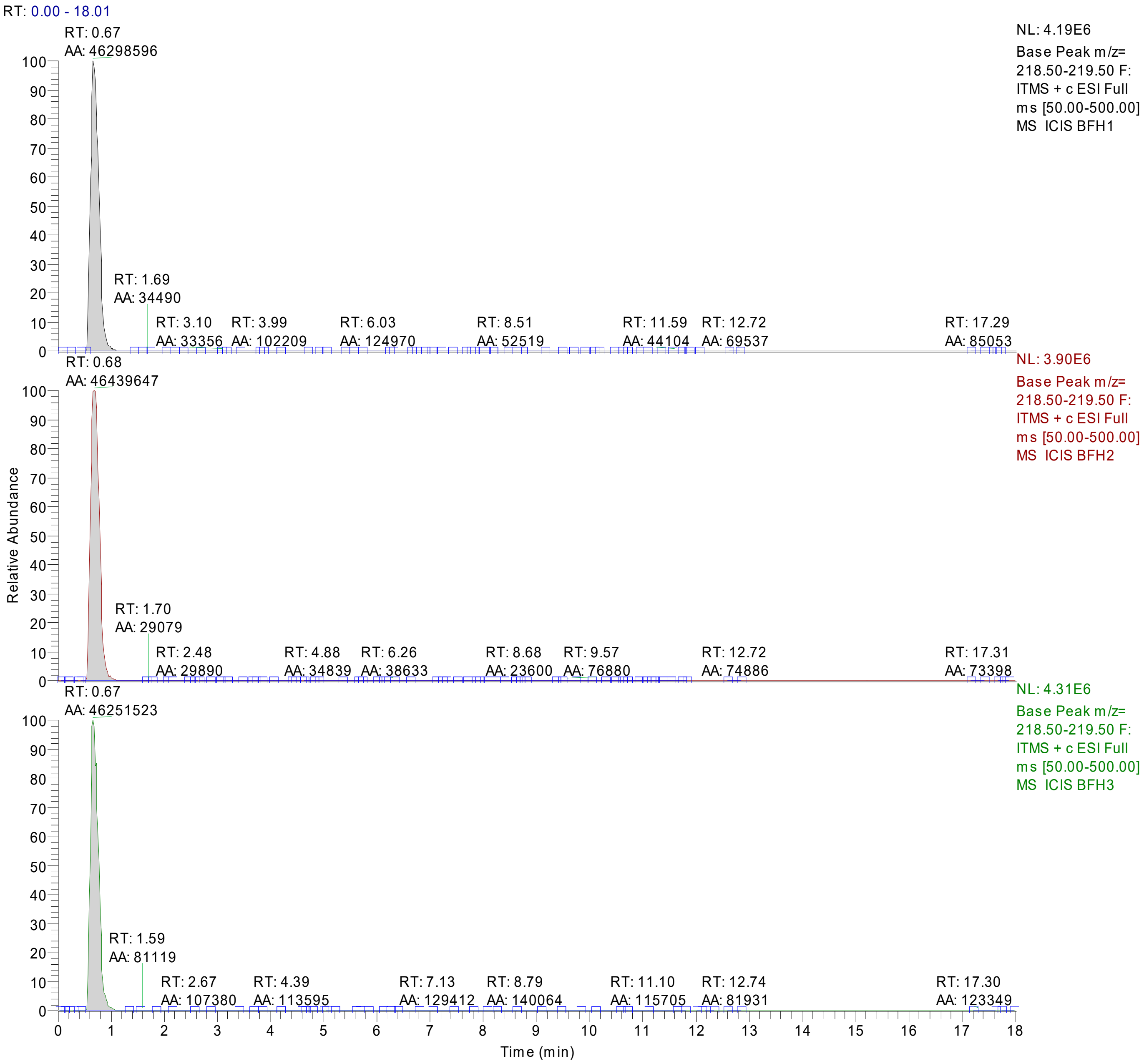

Figure S1. Black Forest honey samples (triplicates): reconstructed ion chromatogram for *m/z* 219 $[M+Na]^+$

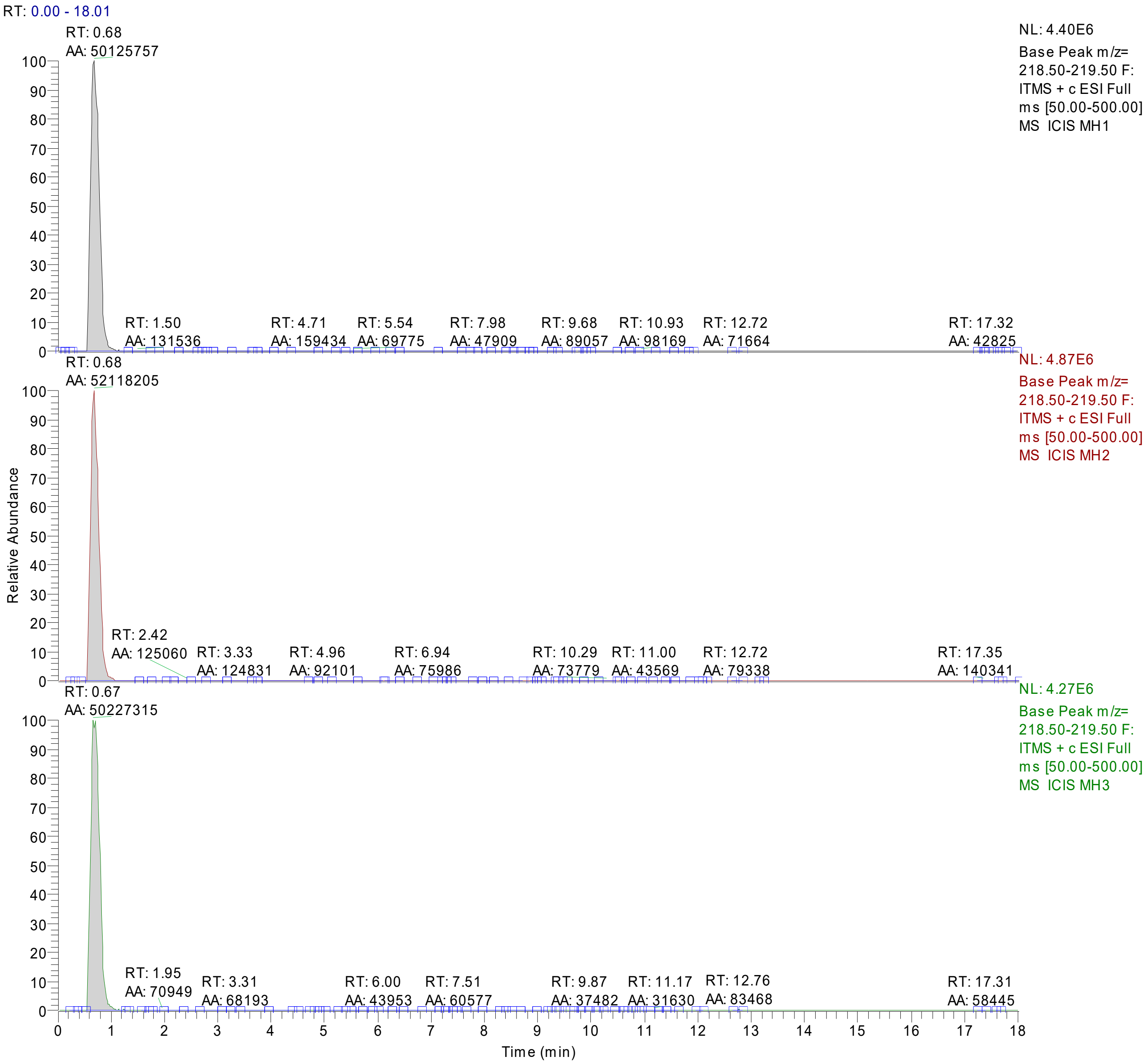

Figure S2. Manuka honey samples (triplicates): reconstructed ion chromatogram for *m/z* 219 $[M+Na]^+$

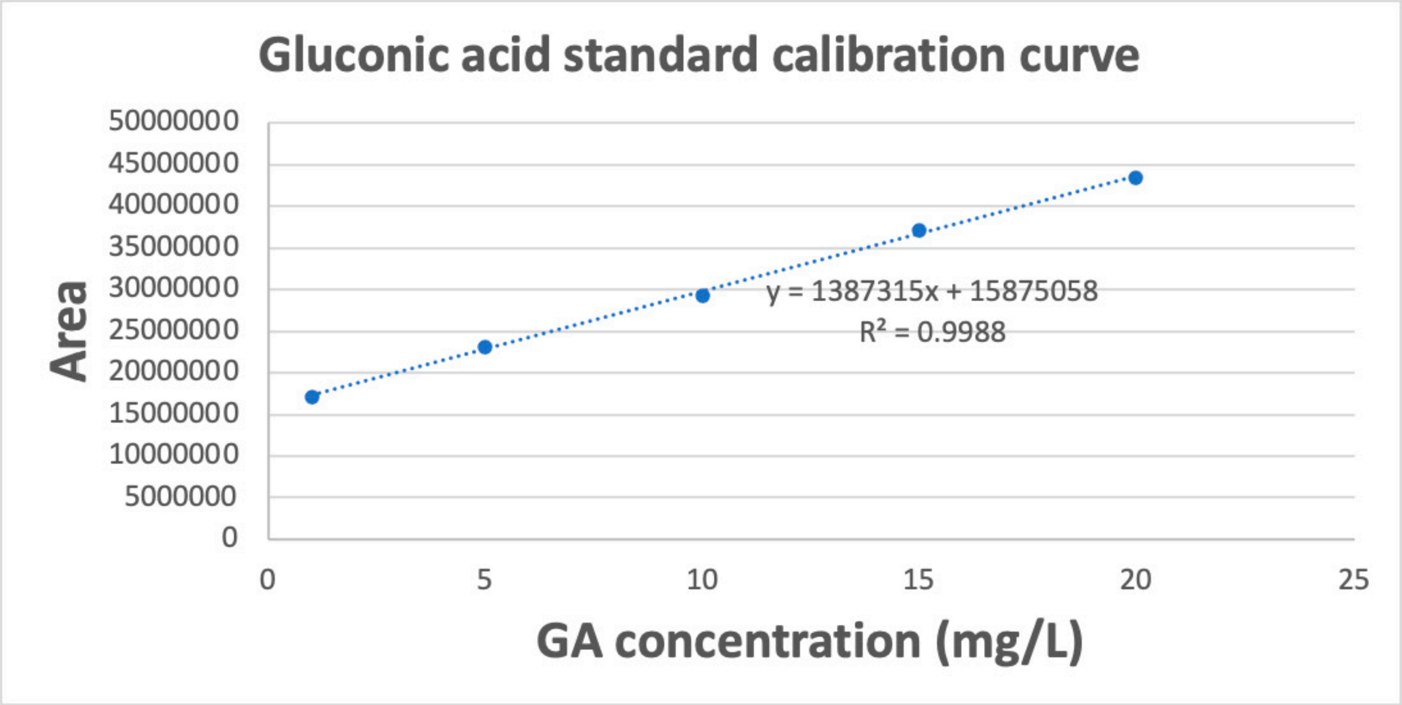

Figure S3. A calibration curve of diluted gluconic acid standard (0, 1, 5, 10, 15, 20 mg/L)
